# Artificial intelligence-based classification of breast lesion from contrast enhanced mammography: a multicenter study

**DOI:** 10.1097/JS9.0000000000001076

**Published:** 2024-01-18

**Authors:** Haicheng Zhang, Fan Lin, Tiantian Zheng, Jing Gao, Zhongyi Wang, Kun Zhang, Xiang Zhang, Cong Xu, Feng Zhao, Haizhu Xie, Qin Li, Kun Cao, Yajia Gu, Ning Mao

**Affiliations:** a Big Data and Artificial Intelligence Laboratory; b Department of Radiology; c Department of Breast Surgery; d Physical Examination Center, Yantai Yuhuangding Hospital, Qingdao University; e School of Computer Science and Technology, Shandong Technology and Business University, Yantai; f Department of Radiology, Weifang Hospital of Traditional Chinese Medicine, Weifang, Shandong; g Department of Radiology, Sun Yat-Sen Memorial Hospital, Sun Yat-Sen University, Guangzhou, Guangdong; h Department of Radiology, Fudan University Shanghai Cancer Center, Shanghai; i Department of Radiology, Beijing Cancer Hospital, Beijing, P. R. China; j Shandong Provincial Key Medical and Health Laboratory of Intelligent Diagnosis and Treatment for Women's Diseases (Yantai Yuhuangding Hospital), Yantai, Shandong, P. R. China

**Keywords:** artificial intelligence, breast, diagnosis, mammography

## Abstract

**Purpose:**

The authors aimed to establish an artificial intelligence (AI)-based method for preoperative diagnosis of breast lesions from contrast enhanced mammography (CEM) and to explore its biological mechanism.

**Materials and methods:**

This retrospective study includes 1430 eligible patients who underwent CEM examination from June 2017 to July 2022 and were divided into a construction set (*n*=1101), an internal test set (*n*=196), and a pooled external test set (*n*=133). The AI model adopted RefineNet as a backbone network, and an attention sub-network, named convolutional block attention module (CBAM), was built upon the backbone for adaptive feature refinement. An XGBoost classifier was used to integrate the refined deep learning features with clinical characteristics to differentiate benign and malignant breast lesions. The authors further retrained the AI model to distinguish *in situ* and invasive carcinoma among breast cancer candidates. RNA-sequencing data from 12 patients were used to explore the underlying biological basis of the AI prediction.

**Results:**

The AI model achieved an area under the curve of 0.932 in diagnosing benign and malignant breast lesions in the pooled external test set, better than the best-performing deep learning model, radiomics model, and radiologists. Moreover, the AI model has also achieved satisfactory results (an area under the curve from 0.788 to 0.824) for the diagnosis of in situ and invasive carcinoma in the test sets. Further, the biological basis exploration revealed that the high-risk group was associated with the pathways such as extracellular matrix organization.

**Conclusions:**

The AI model based on CEM and clinical characteristics had good predictive performance in the diagnosis of breast lesions.

## Introduction

Highlights

- The artificial intelligence (AI) model combining attention-based deep learning features and clinical characteristics can diagnosis of benign and malignant breast lesions, as well as *in situ* and invasive carcinoma and was superior to other deep learning, and radiomics models in the diagnosis of breast lesions.
- The AI model achieved area under the curves of 0.932 for diagnoses of benign and malignant of breast lesions in the pooled external test set, outperforming and improving the diagnostic performance of radiologists.
- The AI prediction was related to pathways such as ECM receptor interactions, focal adhesions, and extracellular matrix organization.

Early diagnosis of breast cancer prior to metastasis allows for increased efficacy in the treatment and consequently leads to notable enhancements in survival rates. In addition, there are significant differences in treatment strategies and prognosis between the two types of breast cancer: in situ and invasive carcinoma. Because of the low incidence of axillary involvement in situ carcinoma (1–2%), sentinel lymph node biopsy (SLNB) is not recommended in planning breast conserving surgery^1^. However, in cases of invasive carcinoma, SLNB or axillary lymph node dissection (ALND) is necessary. Therefore, early diagnosis of *in situ* and invasive carcinoma can develop different surgical and treatment plans for patients.

Contrast enhanced mammography (CEM), as an emerging technology, is increasingly widely used in clinical applications since it can embody the vascularity of the lesions^2^ and has high sensitivity similar to MRI in the diagnosis of breast cancer^3^. Nevertheless, CEM has unsatisfactory specificity (66–84%)^4,5^. Furthermore, the interpretation of traditional imaging examinations can also be influenced by the experience of radiologists because there is great variation among radiologists. In particular, based on these imaging examinations, *in situ* and invasive carcinoma cannot be well distinguished. Therefore, an automatic, reliable, and preoperative non-invasive way to differentiate benign and malignant breast lesions as well as to further differentiate in situ and invasive carcinoma is important.

A powerful artificial intelligence (AI) technology known as “deep learning” is gaining extensive attention for its excellent performance in image recognition tasks^6^. Although previous studies have applied deep learning to CEM images for the prediction of benign and malignant breast lesions^7,8^, the sample size of these studies was small, and they also lacked multicenter data to verify the generalization ability. Moreover, the value of applying deep learning to predict *in situ* and invasive carcinoma on CEM is unclear.

Based on the larger sample size CEM images, previous studies have adopted a deep learning model to differentiate benign and malignant breast lesions, demonstrating satisfactory results^9–11^. Base on this, we further explored the value of CEM images in differentiating in situ and invasive carcinoma in this study. In addition, despite its robust learning capabilities, deep learning lacks the biological interpretability of the learned deep learning features. Few studies have begun to focus on the underlying gene expression patterns of deep learning models^12,13^. However, to our knowledge, there is currently no research focusing on the biological basis of breast cancer prediction models in CEM images.

In this study, we aimed to develop an AI-based classification model that combined deep learning features from CEM images and clinical characteristics for preoperative diagnosis of benign and malignant breast lesions, as well as *in situ* and invasive carcinoma, and used multicenter data from four hospitals for testing. The biological basis under its prediction was also further explored.

## Materials and methods

### Patients and datasets

**Figure 1:**
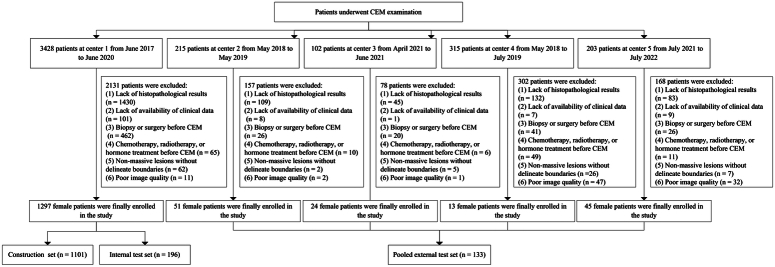
The patient inclusion and exclusion workflow. CEM, contrast enhanced mammography.

Ethical approval for this study was provided by the Ethical Committee of our hospital on 21 November 2022 (Approval No.: 2022-303). The retrospective multicenter study was approved by our institutional review board and patient informed consent was waived. This work has been reported in line with the STARD, Supplemental Digital Content 1, http://links.lww.com/JS9/B719 (Standards for the Reporting of Diagnostic accuracy studies) criteria^14^. Data from five centres from 2017 to 2022 were included. We enroled female patients who underwent CEM examination and histologically confirmed breast lesions from five centres. The patient inclusion and exclusion workflow are shown in Fig. 1 and Supplementary eMethod 1, Supplemental Digital Content 2, http://links.lww.com/JS9/B720. Patients from centre 1 were randomly divided into the construction set (1011), and the internal test set (196), at a ratio of 8.5:1.5. Patients from centres 2–5 were assigned to the pooled external test set (133). The CEM image and clinical characteristics acquisition is shown in Supplementary eMethod 2, Supplemental Digital Content 2, http://links.lww.com/JS9/B720.

### Lesion region segmentation

Lesion regions of interest were manually delineated by R1 on low-energy and recombined images of cranio-cadudal view using ITK-SNAP (version 3.6; www.itksnap.org). The radiologist was blinded to pathological data. The segmentation process is shown in Supplementary eFigure 1, Supplemental Digital Content 2, http://links.lww.com/JS9/B720.

After 4 months, the images of 200 patients were randomly selected and segmented again by another two radiologists with 9 and 13 years of experience in reviewing breast screening (R2 and R3). Then, a dice similarity coefficient (DSC)^15^ was calculated to assess the agreement of the image segmentation.

### Model establishment

**Figure 2:**
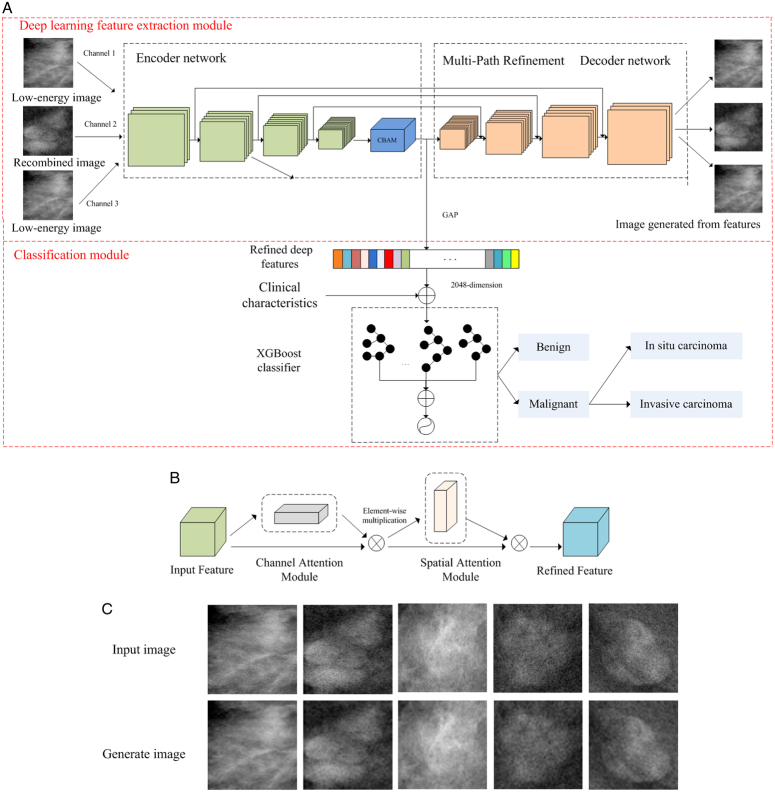
Architecture of our artificial intelligence (AI) model for contrast enhanced mammography (CEM) breast lesions classification. (A) The model includes deep learning feature extraction and classification modules. The deep feature extraction module used RefineNet as the backbone network, and the convolutional block attention module (CBAM) was inserted into the last convolutional layer of the encoder network. The output of CBAM was applied to the global average pooling (GAP) layer to generate refined deep learning features of the CEM images. The classification module used the XGBoost classifier to fuse the refined deep learning features and clinical characteristics for preoperative diagnosis of breast lesions. (B) Network structure of the CBAM attention module. The input feature was sequentially applied to the channel and spatial attention modules to obtain a refined feature. (C) The deep feature extraction module was used to input and generate images.

Figure 2A illustrates the detailed architecture of our proposed AI model. The AI model includes deep learning feature extraction and classification modules. The deep learning feature extraction module used RefineNet^16^ with an encoder and a decoder as the backbone network to extract deep features. Then, a convolutional block attention module (CBAM)^17^ was inserted into the last convolutional layer of the encoder for adaptive features refinement (Fig. 2B). The CBAM is a convolutional attention mechanism module, which can gradually focus on precise targets, namely, high-level semantics. The output by CBAM was applied to the global average pooling (GAP) layer to eliminate redundant features, which were refined deep learning features of the CEM images.

In the classification module, we used the XGBoost classifier to combine the refined deep learning features and clinical characteristics to collaboratively make a decision. The gold standard was pathology-proven based on specimen analysis from either a breast biopsy or surgery, and details can be found in the Supplementary eMethod 3, Supplemental Digital Content 2, http://links.lww.com/JS9/B720. The XGBoost output probability was regarded as the result of benign and malignant classification. To demonstrate the effectiveness of our AI model, ablation experiments were conducted. All details of the model architecture were provided in Supplementary eMethod 4, Supplemental Digital Content 2, http://links.lww.com/JS9/B720. The proposed AI model is also compared with traditional radiomics and clinical models. The detailed construction process of radiomics models is shown in Supplementary eMethod 7, Supplemental Digital Content 2, http://links.lww.com/JS9/B720.

In addition, the proposed AI model was retrained to distinguish *in situ* and invasive carcinoma among breast cancer candidates to further explore the diagnostic value of CEM images.

### Readers study

Additional two radiologists (R4 and R5) with 7 and 13 years of experience in breast screening, who were not involved in other aspects of the study, assessed the benign and malignant nature of breast lesions in the internal and pooled external test sets, respectively. They were blinded to the histopathological data but be aware of the age and CEM images of each patient. The evaluation of benign and malignant breast lesions was based on the following aspects: breast composition, suspicious lesion type (mass, calcification, asymmetry, or architectural distortion), enhancement lesion type, internal enhancement pattern, background parenchymal enhancement, degree of lesion enhancement, and lesion diameter^18,19^. Then, we compared the performance of two radiologists with the AI model.

Each breast lesion was repeatedly diagnosed by radiologists with the AI model assistance to evaluate the assisting ability of the AI model for radiologists. There was no washout period. Finally, the performance of radiologists with the AI model assistance was compared with that of radiologists alone.

### Biological basis exploration

To reveal the underlying biological basis of the AI prediction, gene analyses were performed based on the collected 12 patients with RNA-sequencing data. Twelve patients were divided into high-risk and low-risk subgroups of breast cancer according to AI prediction. The differentially expressed genes (DEGs) of the two risk groups were identified using R package DESeq2 according to the criteria of |log2 (Fold Change)| greater than 2 and adjusted *P* value less than 0.05. Subsequently, Kyoto Encyclopedia of Genes and Genomes (KEGG) and Gene Ontology (GO) analyses were performed using the R package clusterProfiler to identify the enriched pathways between high- and low-risk patients.

### Model evaluation and statistical analysis

The area under the receiver operating characteristic (ROC) curve (AUC), area under the precision-recall curve (AUPRC), and confusion matrix was constructed to assess the performance of the model. The accuracy, sensitivity, specificity, positive predictive value (PPV), and negative predictive value (NPV) were calculated from the ROC curve according to the maximizes of the Youden index, and the corresponding 95% CI was reported. In addition, a 95% sensitivity threshold was defined. Delong’s test^20^ was used to compare the statistical differences between different AUCs. We also evaluated the performance of the model in the different lesion diameter subgroups.

All statistical analyses were implemented using R software (version 4.0.3; www.r-project.org) and Python (version 3.6.6). In clinical characteristics, descriptive statistics were summarized as mean±standard deviation or frequencies and percentages. Continuous variables were used in the independent *t*-test or Manne–Whitney U test, and categorical variables were utilized in the Fisher’s exact test or chi-square test to assess the differences between the patients in different groups. A two-sided *P* less than 0.05 was regarded as the statistically significant difference. Sample size was calculated from a previous pilot reader study^21^.

## Results

### Clinical characteristics

In this study, 1297 patients from centre 1 were divided into a construction set (805 malignant lesions and 296 benign lesions, respectively) and an internal test set (154 malignant lesions and 42 benign lesions, respectively). The 133 patients from centre 2 to centre 5 with 30 benign lesions and 103 malignant lesions were used as a pooled external test set. The mean age was 51.54±11.61 years (range, 17–85 years old) in the construction set, 52.62±11.36 years (range, 17–80 years old) in the internal test set, and 51.23±10.42 years (range, 23–83 years old) in the pooled external test set, respectively. The histopathological type and clinical characteristics are described in Table 1. In addition, 37 of 773 (5%) participants with breast cancer in the construction set, 6 of 148 (4%) in the internal test set, and 10 of 103 (10%) in the pooled external test set were in situ carcinomas. The rest were invasive carcinoma. No statistical differences in clinical characteristics were identified between patients with in situ carcinoma and patients with invasive carcinoma in all datasets (Supplementary eTable 1, Supplemental Digital Content 2, http://links.lww.com/JS9/B720).

**Table 1:** Histopathological type and clinical characteristics for 1430 patients in our dataset.

	Construction set (*n*=1101)			Internal test set (*n*=196)			External test set (*n*=133)		
Parameter	Benign	Malignant	value	Benign	Malignant	value	Benign	Malignant	value
Patients	296 (27)	805 (73)		42 (21)	154 (79)		30 (23)	103 (77)	
Age, years (mean±SD)	43.17±10.98	54.61±10.25	<0.001^*^	42.14±12.53	55.47±9.17	<0.001^*^	44.23±7.00	53.27±10.40	<0.001^*^
Lesion diameter, cm	2.14±1.66	2.49±1.22	<0.001^*^	2.05±2.42	2.54±1.16	0.21	2.23±1.31	3.04±1.81	0.008^*^
≤1	89 (30.1)	35 (4.3)		19 (45.2)	5 (3.3)		3 (10.0)	1 (1.0)	
1–2	99 (33.4)	301 (37.4)		10 (23.8)	51 (33.1)		12 (40.0)	28 (27.2)	
>2	108 (36.5)	469 (58.3)		13 (31)	98 (63.6)		15 (50.0)	74 (71.8)	
Lesion types			—			—			—
Benign lesions									
Fibroadenoma	125 (42.2)	—		20 (47.6)	—		14 (46.7)	—	
Adenosis	70 (23.6)	—		11 (26.2)	—		13 (43.3)	—	
Intraductal papilloma	42 (14.2)	—		4 (9.5)	—		1 (3.3)	—	
Inflammation	9 (3.1)	—		2 (4.8)	—		2 (6.7)	—	
Phyllodes tumour	12 (4.1)	—		0	—		0	—	
Tubular adenoma	0	—		1 (2.4)	—		0	—	
Fibrocystic disease	14 (4.7)	—		1 (2.4)	—		0	—	
Unknow/other	24 (8.1)	—		3 (7.1)	—		0	—	
Malignant lesions									
DCIS	—	30 (3.7)		—	6 (3.9)		—	10 (9.7)	
Intraductal PC	—	7 (0.9)		—	0		—	0	
IDC	—	692 (86.0)		—	135 (87.7)		—	91 (88.3)	
Invasive PC	—	10 (1.2)		—	1 (0.6)		—	0	
ILC	—	17 (2.1)		—	4 (2.6)		—	1 (1.0)	
MAC	—	17 (2.1)		—	2 (1.3)		—	1 (1.0)	
Other	—	32 (4.0)		—	6 (3.9)		—	0	

DCIS, ductal carcinoma *in situ*; IDC, invasive ductal carcinoma; ILC, invasive lobular carcinoma; MAC, mucinous adenocarcinoma; PC, papillary carcinoma.

* *P*<05.

### Segmentation similarity

The average DSC of R1, R2, and R3 was 0.90, of which 0.87 for benign lesions and 0.93 for malignant lesions. R1 and R2 had an average DSC of 0.89. R2 and R3 had an average DSC of 0.93. R1 and R3 had an average DSC of 0.88.

### Model performance

**Figure 3:**
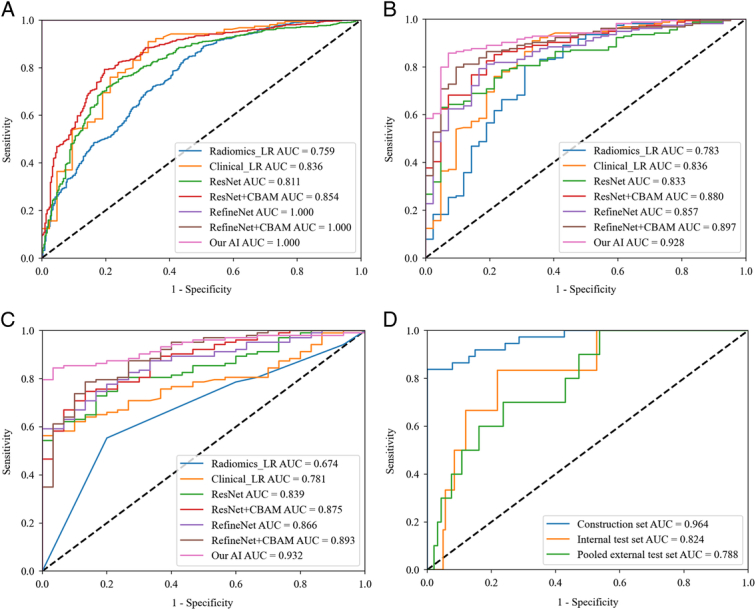
Receiver operating characteristic (ROC) curves of the different models for primary diagnosis of benign and malignant of breast lesions in the construction set (A), internal test set (B), and pooled external test set (C); (D) ROC curves of our AI model for diagnosis of *in situ* and invasive carcinoma among breast cancer candidates in the construction set, internal test set, and pooled external test set. AI, artificial intelligence; AUC, area under the receiver operating characteristic curve; CBAM, convolutional block attention module; LR, logistic regression.

In the pooled external test set, the AI model achieved an AUC of 0.932 (95% CI: 0.891–0.973), higher than those of the RefineNet+CBAM model (AUC: 0.893, 95% CI: 0.831–0.955, *P*=0.20), RefineNet model (AUC: 0.866, 95% CI: 0.804–0.928, *P*=0.06), ResNet+CBAM model (AUC: 0.875, 95% CI: 0.813–0.938, *P*=0.10), ResNet model (AUC: 0.839, 95% CI: 0.771–0.907, *P*=0.005), Clinical model (AUC: 0.781, 95% CI: 0.709–0.841, *P*<0.001), and Radiomics model (AUC: 0.674, 95% CI: 0.593–0.759, *P*<0.001). The performance of different models is summarized in Table 2 and Fig. 3A-C. The AUPRC of the AI model was 0.980 in the internal test set and 0.982 in the pooled external test set, respectively, which was also higher than those of the other models (Supplementary eFigure 2, Supplemental Digital Content 2, http://links.lww.com/JS9/B720).

**Table 2:** Performance of the different models according to construction set, internal and pooled external test sets.

Performance metric	Radiomics_LR	Clinical_LR	ResNet	ResNet+CBAM	RefineNet	RefineNet+CBAM	Our proposed
Construction set							
AUC (95% CI)	0.759 (0.734–0.789)	0.787 (0.760–0.813)	0.811 (0.797–0.823)	0.854 (0.876–0.895)	1.000 (1.000–1.000)	1.000 (1.000–1.000)	1.000 (1.000–1.000)
ACC (95% CI)	0.759 (0.733–0.783)	0.762 (0.738–0.787)	0.724 (0.711–0.735)	0.725 (0.693–0.756)	1.000 (0.999–1.000)	1.000 (0.999–1.000)	1.000 (0.999–1.000)
SENS (95% CI)	0.833 (0.806–0.857)	0.793 (0.763–0.820)	0.717 (0.684–0.747)	0.793 (0.763–0.820)	1.000 (0.994–1.000)	1.000 (0.994–1.000)	1.000 (0.994–1.000)
SPEC (95% CI)	0.554 (0.497–0.610)	0.679 (0.622–0.761)	0.797 (0.746–0.841)	0.804 (0.753–0.847)	1.000 (0.984–1.000)	1.000 (0.984–1.000)	1.000 (0.984–1.000)
PPV (95% CI)	0.837 (0.810–0.861)	0.870 (0.843–0.893)	0.906 (0.880–0.927)	0.917 (0.893–0.936)	1.000 (0.994–1.000)	1.000 (0.994–1.000)	1.000 (0.994–1.000)
NPV (95% CI)	0.547 (0.491–0.603)	0.546 (0.494–0.598)	0.509 (0.462–0.555)	0.588 (0.538–0.636)	1.000 (0.984–1.000)	1.000 (0.984–1.000)	1.000 (0.984–1.000)
*P* value	<0.001	<0.001	<0.001	<0.001	1	1	—
Internal test set							
AUC (95% CI)	0.783 (0.703–0.853)	0.836 (0.770–0.895)	0.833 (0.771–0.900)	0.880 (0.827–0.934)	0.857 (0.798–0.917)	0.897 (0.848–0.946)	0.928 (0.891–0.965)
ACC (95% CI)	0.781 (0.716–0.836)	0.857 (0.800–0.903)	0.699 (0.630–0.762)	0.837 (0.777–0.886)	0.801 (0.738–0.855)	0.821 (0.761–0.872)	0.872 (0.818–0.916)
SENS (95% CI)	0.805 (0.732–0.863)	0.909 (0.849–0.948)	0.630 (0.548–0.705)	0.851 (0.782–0.901)	0.792 (0.718–0.852)	0.798 (0.725–0.857)	0.857 (0.790–0.906)
SPEC (95% CI)	0.690 (0.528–0.819)	0.667 (0.504–0.800)	0.952 (0.826–0.992)	0.786 (0.628–0.892)	0.833 (0.680–0.925)	0.875 (0.701–0.959)	0.929 (0.794–0.981)
PPV (95% CI)	0.905 (0.840–0.946)	0.909 (0.849–0.948)	0.980 (0.922–0.996)	0.936 (0.878–0.968)	0.946 (0.887–0.976)	0.969 (0.916–0.990)	0.978 (0.931–0.994)
NPV (95% CI)	0.492 (0.361–0.622)	0.667 (0.504–0.800)	0.412 (0.315–0.517)	0.589 (0.450–0.716)	0.522 (0.398–0.644)	0.551 (0.427–0.669)	0.639 (0.506–0.755)
*P* value	0.003	0.03	0.001	0.04	0.02	0.15	—
External test set							
AUC (95% CI)	0.674 (0.593–0.759)	0.781 (0.709–0.841)	0.839 (0.771–0.907)	0.875 (0.813–0.938)	0.866 (0.804–0.928)	0.893 (0.831–0.955)	0.932 (0.891–0.973)
ACC (95% CI)	0.609 (0.521–0.692)	0.677 (0.590–0.755)	0.797 (0.719–0.862)	0.767 (0.686–0.836)	0.805 (0.727–0.868)	0.805 (0.727–0.868)	0.857 (0.786–0.912)
SENS (95% CI)	0.553 (0.452–0.650)	0.612 (0.510–0.705)	0.806 (0.714–0.875)	0.757 (0.661–0.834)	0.825 (0.735–0.890)	0.786 (0.692–0.859)	0.903 (0.825–0.950)
SPEC (95% CI)	0.800 (0.609–0.916)	0.900 (0.723–0.974)	0.767 (0.573–0.894)	0.800 (0.609–0.916)	0.733 (0.538–0.870)	0.867 (0.684–0.957)	0.700 (0.504–0.846)
PPV (95% CI)	0.905 (0.798–0.961)	0.955 (0.864–0.988)	0.922 (0.841–0.965)	0.929 (0.845–0.971)	0.914 (0.833–0.959)	0.953 (0.877–0.985)	0.912 (0.835–0.956)
NPV (95% CI)	0.343 (0.236–0.467)	0.403 (0.287–0.530)	0.535 (0.378–0.685)	0.490 (0.346–0.635)	0.550 (0.387–0.704)	0.542 (0.393–0.684)	0.677 (0.485–0.827)
*P* value	<0.001	<0.001	0.005	0.10	0.06	0.20	—

ACC, accuracy; AUC, area under the receiver operating characteristic curve; CBAM, convolutional block attention module; LR, logistic regression; NPV, negative predictive value; PPV, positive predictive value; SENS, sensitivity; SPEC, specificity.

The *P* represents the AUCs differences between each model and our proposed model.

In the pooled external test set, the AI model achieved the best performance with an accuracy of 0.857 (95% CI: 0.796–0.912), sensitivity of 0.903 (95% CI: 0.825–0.950), PPV of 0.912 (95% CI: 0.835–0.956), and NPV of 0.677 (95% CI: 0.485–0.827). The cutoff point maximizing the Youden index and the 95% sensitivity threshold was calculated as 0.89 and 0.35, respectively. The performance of the AI model for 95% sensitivity was shown in Supplementary eFigure 3, Supplemental Digital Content 2, http://links.lww.com/JS9/B720.

For differentiation between in situ and invasive carcinoma, our AI model showed AUCs of 0.824 and 0.788 in the internal test set and pooled external test set; an accuracy of 78.4% and 75.7%; a sensitivity of 83.3% and 70.0%; and a specificity of 78.2% and 76.3% (Fig. 3D and eTable 2, Supplemental Digital Content 2, http://links.lww.com/JS9/B720).

The confusion matrices of the AI model for the diagnosis of breast lesions in two test sets are shown in Supplementary eFigure 4, Supplemental Digital Content 2, http://links.lww.com/JS9/B720. Figure 2C shows the comparison between the input image and the generated image obtained through the AI model, which indicated that the extracted deep learning features contain the inherent features necessary to characterize breast lesions. The accuracy and loss curves of Supplementary eFigure 5, Supplemental Digital Content 2, http://links.lww.com/JS9/B720 show the particle process of the construction set. The figure indicates that the model is well-converged and has no overfitting.

**Figure 4:**
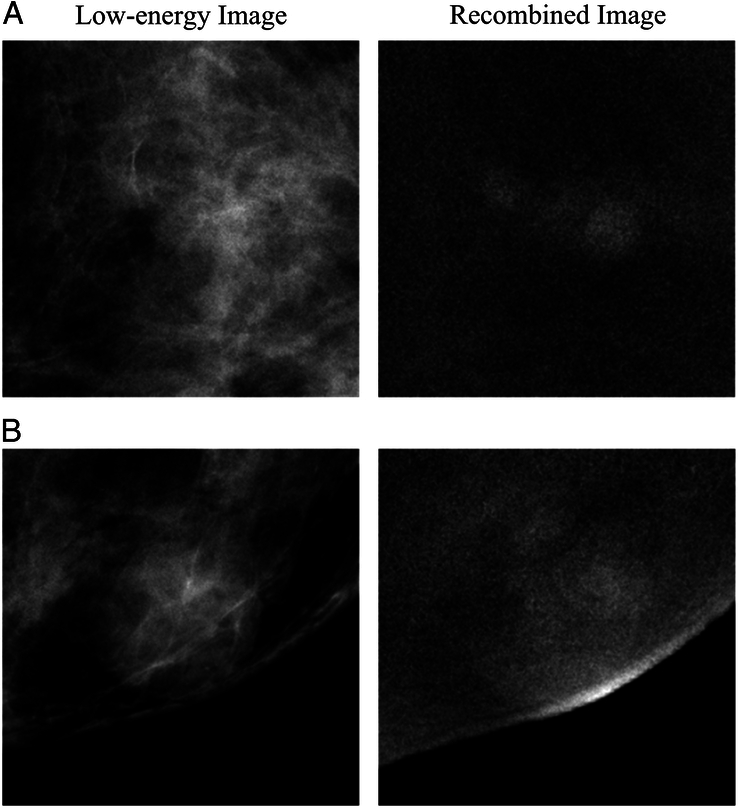
Examples of errors made by the artificial intelligence model. (A) Images in a 56-year-old woman with invasive ductal carcinoma. contrast enhanced mammography (CEM) images show a 0.8 cm mass, BI-RADS 4B. (B) Images in a 56-year-old woman with intraductal papilloma. CEM images show a 1.5 cm mass, BI-RADS 4C.

In addition, we analyzed the wrong cases of the AI model. In the case of Fig. 4A, higher gland density on the low-energy image results in the obscuration of the tumour boundary. In the case of Fig. 4B, heterogeneous enhancement of the lesion and ambiguous boundary lead to the misidentification of AI. AI model has higher error rates for lesions with ambiguous boundaries.

### Comparison with the radiomics and clinical models

The modelling process and model performance of all radiomics models are shown in Supplementary eResult 1. Twenty-seven radiomics features were selected by the three methods of SelectKBest, Pearson correlation, and LASSO (Supplementary eTable 3, Supplemental Digital Content 2, http://links.lww.com/JS9/B720). The ROC curve and performance metric of each radiomics model are shown in Supplementary eTable 4, Supplemental Digital Content 2, http://links.lww.com/JS9/B720 and eFigure 6, Supplemental Digital Content 2, http://links.lww.com/JS9/B720. The radiomics analysis showed that the LR model achieved the best performance in the internal test set (AUC: 0.758, 95% CI: 0.677–0.836) and pooled external test set (AUC: 0.674, 95% CI: 0.593–0.759). The best-performing clinical model was also based on the LR classifier, with the AUCs of 0.836 (95% CI: 0.770–0.895) and 0.781 (95% CI: 0.709–0.841) in the internal and pooled external test sets, respectively (Supplementary eTable 5, Supplemental Digital Content 2, http://links.lww.com/JS9/B720). However, the AI model showed a more favourable performance (Fig. 3 and Table 2).

### Performance of radiologists and radiologists with the AI model assistance

**Figure 5:**
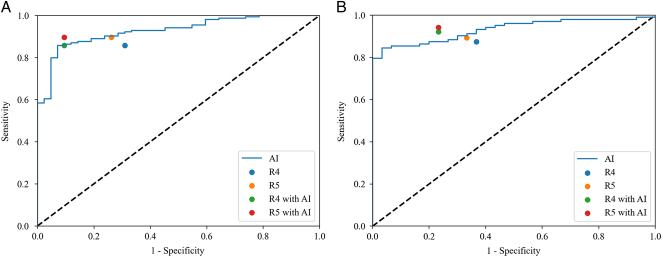
Receiver operating characteristic (ROC) curves of our AI model for diagnosis of benign and malignant of breast lesions and points (specificity and sensitivity) of radiologists in the internal test set (A) and in the pooled external test set (B). AI, artificial intelligence; AUC, area under the ROC curve.

The performance of the AI model was compared with radiologists (R4 and R5) in the two test sets. The specificity and sensitivity points for radiologists’ performance on the internal and pooled external test sets were plotted in the ROC space, as shown in Fig. 5A and B. The figure shows that the points of radiologists lie below the ROC curve of the AI model. Performance metrics are reported in Table 3. In addition, our model spent 1.9 and 1.2 h from segmentation to analyzing imaging on the internal and pooled external test sets, respectively, less time than that of radiologists (Supplementary eTable 6, Supplemental Digital Content 2, http://links.lww.com/JS9/B720).

**Table 3:** Comparisons the performance of the AI model and radiologists for breast lesions identification on internal and pooled external test sets.

	AI	R4	*P*	R5	*P*
Internal test set					
ACC (95% CI)	0.872 (0.818–0.916)	0.821 (0.761–0.872)	0.02	0.862 (0.801–0.905)	0.80
SENS (95% CI)	0.857 (0.790–0.906)	0.857 (0.790–0.906)	>0.99	0.896 (0.834–0.938)	0.01
SPEC (95% CI)	0.929 (0.794–0.981)	0.690 (0.528–0.819)	0.004	0.738 (0.577–0.856)	0.01
PPV (95% CI)	0.978 (0.931–0.994)	0.897 (0.827–0.942)	0.004	0.926 (0.869–0.961)	0.01
NPV (95% CI)	0.639 (0.506–0.755)	0.604 (0.453–0.739)	0.03	0.660 (0.506–0.787)	0.57
Pooled external test set					
ACC (95% CI)	0.857 (0.786–0.912)	0.820 (0.744–0.881)	0.32	0.842 (0.769–0.900)	0.69
SENS (95% CI)	0.903 (0.825–0.950)	0.874 (0.790–0.928)	0.44	0.893 (0.813–0.943)	0.80
SPEC (95% CI)	0.700 (0.504–0.846)	0.633 (0.439–0.795)	0.53	0.667 (0.471–0.821)	0.76
PPV (95% CI)	0.912 (0.835–0.956)	0.891 (0.810–0.942)	0.47	0.902 (0.823–0.949)	0.74
NPV (95% CI)	0.677 (0.485–0.827)	0.594 (0.408–0.758)	0.34	0.646 (0.454–0.802)	0.72

ACC, accuracy; AI, artificial intelligence; NPV, negative predictive value; PPV, positive predictive value; R, radiologist; SENS, sensitivity; SPEC, specificity.

Table 4 illustrates the diagnostic performance of the radiologists with the help of the AI model. Figure 5A and B show that the performance of radiologists with the AI model assistance surpassed our AI model.

**Table 4:** Performance of radiologists with the artificial intelligence (AI) model assistance in internal and pooled external test sets.

	Internal test set		Pooled external test set	
	R4 with AI	R5 with AI	R4 with AI	R5 with AI
ACC (95% CI)	0.867 (0.803–0.911)	0.898 (0.828–0.921)	0.887 (0.821–0.936)	0.902 (0.839–0.947)
SENS (95% CI)	0.857 (0.790–0.906)	0.896 (0.834–0.938)	0.922 (0.848–0.963)	0.942 (0.872–0.976)
SPEC (95% CI)	0.905 (0.765–0.969)	0.905 (0.765–0.969)	0.767 (0.573–0.894)	0.767 (0.573–0.894)
PPV (95% CI)	0.971 (0.922–0.991)	0.972 (0.925–0.991)	0.931 (0.859–0.970)	0.933 (0.861–0.970)
NPV (95% CI)	0.633 (0.498–0.751)	0.704 (0.562–0.816)	0.742 (0.551–0.875)	0.793 (0.597–0.913)

ACC, accuracy; AI, artificial intelligence; NPV, negative predictive value; PPV, positive predictive value; R, radiologist; SENS, sensitivity; SPEC, specificity.

### Performance of the AI model in different lesion diameter subgroups

We tested the performance of the AI model on the different lesion diameter subgroups in the test sets. Supplementary eFigure 7, Supplemental Digital Content 2, http://links.lww.com/JS9/B720 demonstrates that the AUCs of the AI model were 0.811, 0.886, and 0.943 for lesion diameters of less than or equal to 1 cm, 1–2 cm, and greater than or equal to 2 cm subgroups in the internal test set and 1.000, 0.920 and 0.938 in the pooled external test set.

### Biological basis exploration

**Figure 6:**
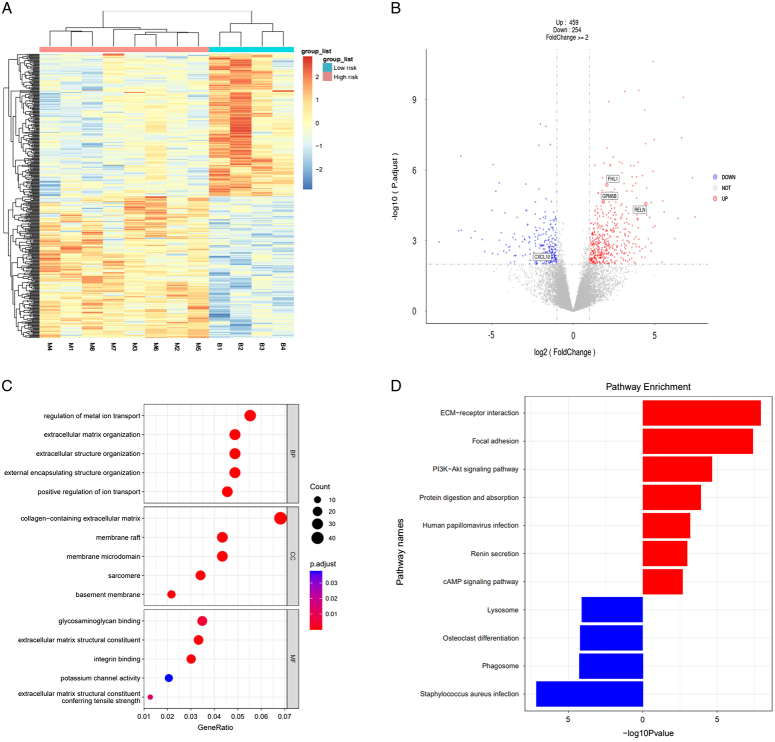
The genetic analysis for exploring the underlying biological basis of the artificial intelligence (AI) model. (A) Heatmap illustrating gene expressions profiles for the 12 breast cancer cases; (B) Volcano diagram of differentially gene expression between risk groups predicted by AI model. The red dots represent genes upregulated in high-risk patients, and the blue dots represent genes upregulated in in low-risk patients; (C) Bubble plot of the top 15 enriched pathways of the differentially expressed gene sets identified by Gene Ontology (GO) analysis, ordered by odds ratio; (D) Upregulated and downregulated pathways in high-risk group based on Kyoto Encyclopedia of Genes and Genomes (KEGG) analysis.

Considering that all breast cancer samples for transcriptome sequencing were invasive carcinomas, we only explored the biological basis in the primary diagnosis model. The heatmap of the genes expression in 8 high-risk patients and 4 low-risk patients is presented in Fig. 6A, demonstrating significant differences between the two risk groups. Simultaneously, the differentially expressed genes related to breast cancer, such as FHL1, GPM6B, RELN, and CXCL10 were discovered between the two risk groups (Fig. 6B). The KEGG and GO analyses based on the DEGs identified the key biological pathway, as shown in Fig. 6C and D. KEGG analysis inferred that several pathways such as ECM−receptor interaction, focal adhesion, and human papillomavirus infection were significantly upregulated in high-risk patients. From the enriched pathways based on the GO analysis, we found that these pathways were mainly enriched in the extracellular matrix organization and structure organization.

## Discussion

In this multicenter study, we developed and tested an AI model combining deep learning features and clinical characteristics for the preoperative diagnosis of breast lesions on CEM. Compared with other models, the AI model demonstrated the best prediction performance, with an AUC of 0.932 on the pooled external test set. Notably, an association analysis of AI prediction with RNA-sequencing was performed to reveal the underlying biological basis of the model. To the best of our knowledge, this is the first study to distinguish *in situ* and invasive carcinoma based on AI on CEM images and explore the biological basis of an AI model.

Several studies have shown that radiomics can predict benign and malignant breast lesions^5,22–24^. However, the features extracted by this method are manually designed and have great instability. The deep learning method can not only automatically extract high-throughput features, avoiding the bias of manual design features in radiomics; and it can achieve superior performance compared with traditional methods with the increase in the amount of training data^25^. In this study, we also demonstrated that the AI model surpassed radiomics models. Perek *et al.* ^7^ first presented a deep learning decision support system based CEM images to improve the specificity (66%) of breast cancer diagnosis without affecting sensitivity, but only 129 patients were included. Song *et al.* ^8^ proposed a multiview multimodal network for breast cancer diagnosis on CEM, wherein only 95 patients were enroled. Dominique *et al.* ^26^ presented a deep learning model that effectively characterizes breast malignant lesions, which demonstrated a good performance to characterize oestrogen receptor status and to differentiate triple-negative breast cancers. However, no external verification was conducted. These studies may not meet the actual requirements because the large amount and multicenter data were not yet available. Thus, comparing with previous similar studies, we collected a larger data set and used multicenter data from various regions of China (east, west, south, and north) to evaluate the generalization and application capabilities of our model. Many studies have also begun to focus on the prediction of breast ductal carcinoma *in situ* using MRI or ultrasound^27,28^, but the differential value of CEM images in distinguishing in situ carcinoma from invasive carcinoma is still unclear. In the present study, we have found that CEM images had a good performance for the discrimination of in situ and invasive carcinoma based on AI model.

Recently, many studies has achieved good results in deep learning-based diagnosis of benign and malignant breast lesions with CEM images^9–11,29^. In contrast, in this study, we proposed an AI model that can not only distinguish benign and malignant breast lesions but also differentiate *in situ* and invasive carcinoma. In addition, we incorporated data from more centres for model generalization ability testing. Notably, before applying AI models based on imaging in clinical settings, it is crucial to elucidate the underlying biological basis behind them^30^. To move forward, we employed gene analysis of RNA-sequencing data to uncover the biological foundation of the AI model. The results showed that the high-risk phenotypes were linked to the tumour proliferation pathway such as ECM−receptor interaction, extracellular matrix organization, and focal adhesion. The extracellular matrix could influence the invasion of tumour cells through different mechanical signal transduction pathways^31^. A core component of the focal adhesion pathway is focal adhesion kinase, which can regulate nucleostemin levels, a nucleolar protein involved in promoting breast tumour growth^32^. This is consistent with our findings.

We defined the cutoff point maximizing the Youden index and the 95% sensitivity threshold. The cutoff point maximizing the Youden index balanced the sensitivity and specificity of the model, resulting in the highest accuracy (85–87%) in diagnosing benign and malignant lesions. The 95% sensitivity threshold could achieve an NPV of 73–76%, thus the AI model could prevent patients with benign breast lesions from undergoing unnecessary biopsies on the premise of avoiding missed diagnosis.

Our study also has several limitations. First, we included a sizeable and multicenter CEM dataset but did not include prospective and international datasets, and also excluded non-mass lesions. We will focus on collecting a larger variety of lesion types and more cross-regional and cross-country datasets in future prospective studies to further improve the generalization and application capabilities of the model. Second, although the dice similarity coefficient achieved good agreement, the segmentation of the lesions was manually drawn by the radiologists. Third, the sample size of benign and malignant lesions in the dataset is unbalanced. Although the class weight method was used to mitigate this problem, the class-imbalanced problem may not be completely solved. Fourth, background parenchymal enhancement may impact the model performance in small lesions. Fifth, the study only included low-energy and recombined images of the cranio-cadudal view. Mediolateral-oblique view, high-energy images, and other modal images will also be included in the analysis to add more image information. Lastly, we only collected RNA-sequencing data from 12 cases and will continue to expand the sample size for further exploration.

In conclusion, the AI model we proposed that integrated refined deep learning features and clinical characteristics can efficiently and noninvasively identify benign and malignant breast lesions from CEM, and distinguish in situ from invasive carcinoma. Further prospective and different countries verifications in the future will provide additional evidence for our model to assist clinical decision-making.

## Ethical approval

Ethical approval for this study was provided by the Ethical Committee of Yantai Yuhuangding.

Hospital on 21 November 2022 (Approval No.: 2022-303).

## Consent

The retrospective multicenter study was approved by the Yantai Yuhuangding Hospital institutional review board and patient informed consent was waived.

## Source of funding

This study was supported by the National Natural Science Foundation of China (82371933, 82001775 and 62176140), Taishan Scholar Foundation of Shandong Province of China (tsqn202211378), Natural Science Foundation of Shandong Province of China (ZR2021MH120) and Special Fund for Breast Disease Research of Shandong Medical Association (YXH2021ZX055)

## Author contribution

H.Z., and F.L. are co–first authors. N.M. had full access to all of the data in the study and takes responsibility for the integrity of the data and the accuracy of the data analysis. H.Z. and N.M. contributed to concept and design of study. All authors contributed to acquisition, analysis, or interpretation of data. N.M., Y.G., and K.C. provided supervision. H.Z. developed the study methodology and performed the statistical analyses. H.Z. and F.L. drafted the manuscript. All authors performed critical revision of the manuscript for important intellectual content. N.M., H.X. and F.Z. contributed to funding acquisition. All authors read and approved the final manuscript.

## Conflicts of interest disclosure

None.

## Research registration unique identifying number (UIN)

Chinese Clinical Trial Registry.

ChiCTR2200063444.

https://www.chictr.org.cn/showproj.html?proj=173057.

## Guarantor

Ning Mao and Haicheng Zhang are the guarantors of this paper.

## Data availability statement

Some or all data, models, or code generated or used during the study are available from the corresponding author by request.

## Provenance and peer review

This paper was not invited.

## Supplementary Material

## References

[1] Gradishar WJ Moran MS Abraham J. Breast Cancer, Version 3.2022, NCCN Clinical Practice Guidelines in Oncology. J Natl Compr Canc Netw 2022;20:691–722. 35714673 10.6004/jnccn.2022.0030

[2] Patel BK Lobbes MBI Lewin J. Contrast enhanced spectral mammography: a review. Semin Ultrasound CT MR 2018;39:70–79. 29317041 10.1053/j.sult.2017.08.005

[3] Lee-Felker SA Tekchandani L Thomas M. Newly diagnosed breast cancer: comparison of contrast-enhanced spectral mammography and breast MR imaging in the evaluation of extent of disease. Radiology 2017;285:389–400. 28654337 10.1148/radiol.2017161592

[4] Marino MA Pinker K Leithner D. Contrast-enhanced mammography and radiomics analysis for noninvasive breast cancer characterization: initial results. Mol Imaging Biol 2020;22:780–787. 31463822 10.1007/s11307-019-01423-5 PMC7047570

[5] Patel BK Ranjbar S Wu T. Computer-aided diagnosis of contrast-enhanced spectral mammography: a feasibility study. Eur J Radiol 2018;98:207–213. 29279165 10.1016/j.ejrad.2017.11.024

[6] LeCun Y Bengio Y Hinton G. Deep learning. Nature 2015;521:436–444. 26017442 10.1038/nature14539

[7] Perek S Kiryati N Zimmerman-Moreno G. Classification of contrast-enhanced spectral mammography (CESM) images. Int J Comput Assist Radiol Surg 2019;14:249–257. 30367322 10.1007/s11548-018-1876-6

[8] Song J Zheng Y Zakir Ullah M. Multiview multimodal network for breast cancer diagnosis in contrast-enhanced spectral mammography images. Int J Comput Assist Radiol Surg 2021;16:979–988. 33966155 10.1007/s11548-021-02391-4

[9] Mao N Zhang H Dai Y. Attention-based deep learning for breast lesions classification on contrast enhanced spectral mammography: a multicentre study. Br J Cancer 2023;128:793–804. 36522478 10.1038/s41416-022-02092-y PMC9977865

[10] Zheng T Lin F Li X. Deep learning-enabled fully automated pipeline system for segmentation and classification of single-mass breast lesions using contrast-enhanced mammography: a prospective, multicentre study. EClinicalMedicine 2023;58:101913. 36969336 10.1016/j.eclinm.2023.101913 PMC10034267

[11] Chen Y Hua Z Lin F. Detection and classification of breast lesions using multiple information on contrast-enhanced mammography by a multiprocess deep-learning system: a multicenter study. Chinese J Cancer Res 2023;35:408–423. 10.21147/j.issn.1000-9604.2023.04.07 PMC10485921 37691895

[12] She Y He B Wang F. Deep learning for predicting major pathological response to neoadjuvant chemoimmunotherapy in non-small cell lung cancer: a multicentre study. EBioMedicine 2022;86:104364. 36395737 10.1016/j.ebiom.2022.104364 PMC9672965

[13] Yan J Zhao Y Chen Y. Deep learning features from diffusion tensor imaging improve glioma stratification and identify risk groups with distinct molecular pathway activities. EBioMedicine 2021;72:103583. 34563923 10.1016/j.ebiom.2021.103583 PMC8479635

[14] Bossuyt PM Reitsma JB Bruns DE. STARD 2015: an updated list of essential items for reporting diagnostic accuracy studies. BMJ 2015;351:h5527. 26511519 10.1136/bmj.h5527 PMC4623764

[15] Pereira S Pinto A Alves V. Brain tumor segmentation using convolutional neural networks in MRI images. IEEE Trans Med Imaging 2016;35:1240–1251. 26960222 10.1109/TMI.2016.2538465

[16] Lin G Liu F Milan A. RefineNet: Multi-path refinement networks for dense prediction. IEEE Trans Pattern Anal Mach Intell 2020;42:1228–1242. 30668461 10.1109/TPAMI.2019.2893630

[17] Woo S Park J Lee J-Y. (2018) CBAM: Convolutional Block Attention Module. arXiv:1807.06521.

[18] Wang S Sun Y Mao N. Incorporating the clinical and radiomics features of contrast-enhanced mammography to classify breast lesions: a retrospective study. Quant Imaging Med Surg 2021;11:4418–4430. 34603996 10.21037/qims-21-103 PMC8408786

[19] D’Orsi CJSE Mendelson EB Morris EA. ACR BI-RADS® Atlas, Breast Imaging Reporting and Data System. American College of Radiology; 2013.

[20] DeLong ER D DM Clarke-Pearson DL. Comparing the areas under two or more correlated receiver operating characteristic curves: a nonparametric approach. Biometrics 1988;44:837–845. 3203132

[21] Obuchowski NA. Sample size tables for receiver operating characteristic studies. AJR Am J Roentgenol 2000;175:603–608. 10954438 10.2214/ajr.175.3.1750603

[22] Zhang Q Peng Y Liu W. Radiomics based on multimodal MRI for the differential diagnosis of benign and malignant breast lesions. J Magn Reson Imaging 2020;52:596–607. 32061014 10.1002/jmri.27098

[23] Fleury E Marcomini K. Performance of machine learning software to classify breast lesions using BI-RADS radiomic features on ultrasound images. Eur Radiol Exp 2019;3:34. 31385114 10.1186/s41747-019-0112-7 PMC6682836

[24] Li H Mendel KR Lan L. Digital mammography in breast cancer: additive value of radiomics of breast parenchyma. Radiology 2019;291:15–20. 30747591 10.1148/radiol.2019181113 PMC6445042

[25] Xi IL Zhao Y Wang R. Deep learning to distinguish benign from malignant renal lesions based on routine MR imaging. Clin Cancer Res 2020;26:1944–1952. 31937619 10.1158/1078-0432.CCR-19-0374

[26] Dominique C Callonnec F Berghian A. Deep learning analysis of contrast-enhanced spectral mammography to determine histoprognostic factors of malignant breast tumours. Eur Radiol 2022;32:4834–4844. 35094119 10.1007/s00330-022-08538-4 PMC8800426

[27] Hou R Grimm LJ Mazurowski MA. Prediction of upstaging in ductal carcinoma in situ based on mammographic radiomic features. Radiology 2022;303:54–62. 34981975 10.1148/radiol.210407 PMC8962778

[28] Zhu M Pi Y Jiang Z. Application of deep learning to identify ductal carcinoma in situ and microinvasion of the breast using ultrasound imaging. Quant Imaging Med Surg 2022;12:4633–4646. 36060588 10.21037/qims-22-46 PMC9403599

[29] Beuque MPL Lobbes MBI van Wijk Y. Combining deep learning and handcrafted radiomics for classification of suspicious lesions on contrast-enhanced mammograms. Radiology 2023;307:e221843. 37338353 10.1148/radiol.221843

[30] Bi WL Hosny A Schabath MB. Artificial intelligence in cancer imaging: clinical challenges and applications. CA Cancer J Clin 2019;69:127–157. 30720861 10.3322/caac.21552 PMC6403009

[31] Winkler J Abisoye-Ogunniyan A Metcalf KJ. Concepts of extracellular matrix remodelling in tumour progression and metastasis. Nat Commun 2020;11:5120. 33037194 10.1038/s41467-020-18794-x PMC7547708

[32] Tancioni I Miller NL Uryu S. FAK activity protects nucleostemin in facilitating breast cancer spheroid and tumor growth. Breast Cancer Res 2015;17:47. 25880415 10.1186/s13058-015-0551-x PMC4407832

