# Supplementary material for: Artificial intelligence-based classification of breast lesion from contrast enhanced mammography: a multicenter study
Source: Int J Surg. 2024 Jan 18;110(5):2593–603. doi: 10.1097/JS9.0000000000001076 (PMC11093474; doi:10.1097/JS9.0000000000001076)
Supplement: Supplementary file 2 [file js9-110-2593-s002.docx]

**Supplementary Materials**

**eMethod 1 Patient inclusion and exclusion workflow**

The inclusion criteria were as follows: (1) patients with suspected breast lesions after physical examination, mammography, or ultrasound; (2) patients who were referred for CEM as part of diagnostic imaging; (3) clear pathological results of breast lesions; (4) availability of clinical information; and (5) CEM images within 2 weeks before biopsy or surgery. The exclusion criteria were as follows: (1) patients underwent biopsy or surgery before CEM examination; (2) patients underwent chemotherapy, radiotherapy, or hormone treatment before CEM examination; (3) non-mass lesions without delineated boundaries; and (4) poor image quality. For the image quality evaluation, it was judged by a senior and a junior radiologist together. Additionally, the largest lesion was selected for patients with multifocal breast lesions [1].

**eMethod 2 CEM Image and Clinical Characteristics Acquisition**

The CEM data from center 1 were collected with a Senographe Essential full-digital breast machine of the GE Healthcare Company, while CEM data from center 2 were obtained with a Senographe Pristina full-digital breast machine of the GE Healthcare Company. The imaging process of both centers was consistent. The patient’s upper arm vein was injected with the contrast agent iopamidol (300 mg/ml, GE Healthcare, Inc., Princeton, NJ) using a high-pressure syringe, with a dose of 1.5 ml/kg and injection flow rate of 1.5 ml/s. The low-energy and high-energy images of the cranial caudal (CC) and mediolateral oblique (MLO) view were acquired 2 min after injection. Eight images of both sides were collected within 5 minutes. The recombined images were obtained by subtracting the high- and low- energy image.

**eMethod 3 Ground truth**

The gold standard for the pathological diagnosis of eligible patients is histopathology, which is determined through the pathological examination of biopsy or surgical resection specimens. In this examination, atypia in breast tumor tissues and cells is a manifestation of maturation and differentiation disorders, thus serving as a crucial indicator to differentiate between benign and malignant tumors. Benign tumors exhibit less atypia, whereas malignant tumors display greater atypia. Although the cellular atypia of benign tumors is minimal, varying degrees of structural atypia still exist. Malignant tumors showcase notable cellular and structural atypia. A higher degree of atypia corresponds to reduced maturity and differentiation of tumor tissue and cells, and a greater dissimilarity compared to corresponding normal tissues.

Breast cancer exhibits a complex histomorphology and is classified into two main categories: in situ and invasive carcinoma. In situ carcinoma implies that cancer cells are confined within the breast duct or gland, without breaching the basement membrane into the surrounding tissues. On the other hand, invasive carcinoma refers to the infiltration of cancer cells into the normal structure of breast tissue, where they penetrate the basement membrane and spread into the surrounding tissues.

**eMethod 4 Model Establishment**

A 2D binary mask was created from the delineated lesion region of interest (ROI) for each breast lesion (Supplementary eFigure 1). The mask was circumferentially dilated by 40 pixels to encompass tissue adjacent to the breast lesion. A rectangular box was cropped from the original image according to the size of the mask. The ROI was centered within the rectangular box. Then, all the cropped images were resized to 224×224 pixels for the network inputs and processed max-min normalization. To alleviate the over-fitting problem that may be caused by the limited data set, data augmentation techniques with random flipping, rotation, and scaling were applied to the training set.

The AI model includes deep feature extraction and classification modules (Figure 2a). The deep feature extraction module used RefineNet [2] with an encoder and a decoder as the backbone network to extract deep features. The encoder network utilized the ResNet101 [3] pre-trained on the ImageNet dataset [4] to extract the deep features of the input image. The number of filters in each layer of the encoder was set to 256, 512, 1024, and 2048. The decoder used the deep features of each layer to reconstruct the input breast lesion image to evaluate the performance of deep features. The structural details of RefineNet network are provided in Supplementary eMethod 5. Then, a convolutional block attention module (CBAM) [5] was inserted into the last convolutional layer of the encoder for adaptive features refinement. The CBAM is a convolutional attention mechanism module, which can gradually focus on precise targets, namely, high-level semantics. The deep features generated by the backbone network were sequentially applied to the channel and spatial attention modules to obtain the refined deep features. The network structure of the CBAM is show in Figure 2b and Supplementary eMethod 6. The output by CBAM was applied to the global average pooling (GAP) layer to eliminate redundant features, which were refined deep features of the CEM images with 2048 dimensions.

In the classification module, we used the XGBoost classifier to combine the refined deep features and clinical characteristics to collaboratively make a decision. The XGBoost output probability was regarded as the result of benign and malignant classification. Then, the proposed AI model was further used to explore the value in the diagnosis of in situ and invasive carcinoma among breast cancer candidates.

The construction set were used fivefold cross-validation to evaluated model and select optimal hyperparameters for the final trained models. Meanwhile, the internal and pooled external test sets were used to test the model. Considering that the input to the pre-trained model is a three-channel image, a three-channel image consisting of the low-energy image, recombined image and low-energy image of each patient were fed into the model. The Stochastic Gradient Descent optimizer was used to update the model parameters using a batch size of 12 and a learning rate of 0.0001. The weights of the pre-trained model were used as the initialization weights of the model. To mitigate the problem of class imbalance between benign and malignant samples, the ‘class weight’ method in the sklearn.utils python package was used. The basic principle is to directly modify the loss function by penalizing classes with different weights, purposefully increasing the weight of the minority class and reducing the weight of the majority class. The loss of the model was calculated using the MSE loss function. After 50 epochs, the model with the best validation loss and accuracy was selected. The XGBoost was trained with a learning rate of 0.59, an n_estimator of 500, a max_deep of 4, and a scale_pos_weight of 3.8. The proposed model was implemented on Keras platform (version 2.4.3; https://keras.io/) with TensorFlow 1.6.0 as the backend and was trained with 2× NVIDIA GeForce TITAN RTX GPU (24 GB).

To demonstrate the effectiveness of our AI model, ablation experiments were conducted. First, we built a model that only used CEM images (RefineNet+CBAM) to compare with the AI model. Second, the combination of attention-based RefineNet network and XGBoost (RefineNet+CBAM) was replaced to the end-to-end attention-based ResNet101 network (ResNet+CBAM) to explore the effectiveness of using RefineNet for feature extraction, and the others remain the same to ensure fair comparison. Lastly, the CBAM was deleted from the attention-based RefineNet and attention-based ResNet101, denoted as RefineNet and ResNet, respectively, to prove the necessity of attention mechanism. The performance of different models is summarized in Table 2.

**eMethod 5 RefineNet Network**

RefineNet network consisted of a down-sampling encoder network and an up-sampling decoder network. The encoder network extracted deep features of the input image using ResNet101 as the fundamental building block. The network contained four downsampling operations, corresponding to the four layers of ResNet. The number of filters in each layer was set to 256, 512, 1024, and 2048. Specifically, the encoder network finally transformed a breast lesion image into a 2048-dimensional mineable feature vector.

The decoder network used the deep features of each layer to reconstruct the input breast lesion image to evaluate the performance of deep features. This network used the remote residual connection and four up-sampling operations to restore feature maps with multiple resolutions to the original image information. First, two of Rectified Linear Unit and 3$\times$3 convolutional (Conv) operations (Residual Convolution Unit [RCU]) were used to obtain feature maps of constant size obtained by downsampling. Then, the feature maps of different resolutions were fused using 3$\times$3 Conv layers and upsampling operations. Next, the image context information was used the chain the residual pooling structure for maximum range extraction. Finally, after two of RCU, the softmax activation function was applied to obtain the final prediction result. If the image reconstructed by the decoder network is closer to the input lesion image, then the deep features extracted from breast lesions are more effective. The minimum mean square error (MSE) was required to compare the similarity between the restored image and the original image.

**eMethod 6 Convolutional Block Attention Module**

CBAM is a convolutional attention mechanism module. It include channel attention and spatial attention module. The network structure of the CBAM attention module is show in Figure 2b. The channel attention module is focused on what input features are meaningful. The input features are simultaneously input to the average and max pooling layers to aggregate the spatial information of the feature map. Then, the generated descriptors are sent to the multi-layer perceptron network to get the two feature maps. The channel attention map obtained by the element-wise summation and sigmoid operation of these two feature maps are element-wise multiplied by the input feature to obtain the new feature map. The spatial attention module is focused on were input features are meaningful. The new feature map is applied average and maximum pooling operations, and connected to generate a descriptor. Then it is applied to a 7×7 convolution kernel and sigmoid operation, and finally element-wise multiplied by the new feature map to get the refined feature.

**eMethod 7 Radiomics and Clinical Models Construction**

Image preprocessing, including image normalization and image resampling, was needed before feature extraction. The image was resampled to 0.1 mm × 0. 1 mm to ensure the scale invariance of features. Image normalization improved the texture feature differences by adopting the μ ± 3σ method. The gray values greater than μ ± 3σ were set to nan. Radiomics features were extracted from each CEM image with manually segmented ROI using PyRadiomics python package. The extracted features included three categories: first-order statistics, shape features, and texture features. The first-order statistics include Energy, Entropy, InterquartileRange, and etc. The shape statistics include Elongation, Flatness, SphericalDisproportion, and etc. The texture features include gray-level cooccurrence matrix (GLCM), gray-level run length matrix (GLRLM), gray-level size zone matrix (GLSZM), gray-level dependence matrix (GLDM), and neighboring gray tone difference matrix (ngtdm). The original image was also processed by Laplacian Gaussian filtering, wavelet, and other filters to generate filtered images. A total of 1409 radiomics features were extracted for each CEM image based on the original and filtered images. Accordingly, each patient were extracted a total of 1409×2 radiomics features from the low-energy image and the recombined image. All the features were normalized using Z-Score normalization, and inter- and intra-correlation coefficients (ICCs) were used to evaluate the intra-/inter-reader reproducibility of the radiomics features. The next analysis considered radiomics features of ICCs > 0.75.

To reduce dimensionality of the radiomics features, the feature selection methods were as follows: (1) The SelectKBest method was used to calculate the significance level of differentiating benign and malignant lesions and select features with significant differences (p < 0.05). (2) The Pearson correlation was used to calculate the correlation (denoted as “r” hereafter) of each feature pairs. If |r| > 0.9, then one of the features is removed. (3) The least absolute shrinkage and selection operator (LASSO) algorithm, the optimal alpha penalty operator and non-zero coefficient conducted by five-fold cross-validation, was applied to obtain the final features.

Multiple traditional radiomics models, namely, logistic regression, support vector machine, K-Nearest Neighbor, and decision tree were built based on the selected radiomics features. We also used these four classifiers to build multiple clinical models based on clinical characteristics for comparison with the proposed AI model.

**eResult 1 Performance of Radiomics Models**

The inter- and intra-observer reproducibility of extracting radiomics features was robust, and the ICCs reached 0.75 in between the two different readers and the same reader. Twenty-seven radiomics features were selected by the three methods of SelectKBest, Pearson correlation, and LASSO (eTable 3). Multiple traditional radiomics models, namely, LR, SVM, KNN, and DT, were built on the basis of the selected radiomics features. The ROC curve and performance metric of each radiomics model are shown in eTable 4 and eFigure 6. The LR classifier achieved the best performance in predicting the classification of benign and malignant breasts on the pooled external test set (AUC: 0.715; 95% CI: 0.619–0.817 higher than with AUCs of 0.641 (95% CI: 0.527–0.771) for SVM, 0·644 (95% CI: 0.549–0.763) for KNN, and 0.651 (95% CI: 0.536–0.781) for DT.

**eReference**

1. Chen H, Yang BW, Qian L, Meng YS, Bai XH, Hong XW, et al. Deep Learning Prediction of Ovarian Malignancy at US Compared with O-RADS and Expert Assessment. Radiology. 2022;304(1):106-13.

2. Lin G, Liu F, Milan A, Shen C, Reid I. RefineNet: Multi-Path Refinement Networks for Dense Prediction. IEEE Trans Pattern Anal Mach Intell. 2020;42(5):1228-42.

3. He K, Zhang X, Ren S, Sun J. Deep Residual Learning for Image Recognition2015 December 01, 2015:[arXiv:1512.03385 p.]. Available from: <https://ui.adsabs.harvard.edu/abs/2015arXiv151203385H>.

4. Olga Russakovsky JD, Hao Su, Jonathan Krause, Sanjeev Satheesh, Sean Ma, Zhiheng Huang, Andrej Karpathy, Aditya Khosla, Michael Bernstein, Alexander C. Berg, Li Fei-Fei. ImageNet Large Scale Visual Recognition Challenge. International Journal of Computer Vision. 2015;115:211–52.

5. Woo S, Park J, Lee J-Y, Kweon IS. CBAM: Convolutional Block Attention Module2018 July 01, 2018:[arXiv:1807.06521 p.]. Available from: <https://ui.adsabs.harvard.edu/abs/2018arXiv180706521W>.

**Supplementary Table**

**eTable 1** Clinical characteristics for 1024 patients of breast cancer in our dataset.

|  | **Construction Set (n=773)** | | | **Internal Test Set (n=148)** | | | **External Test Set (n=103)** | | |
| --- | --- | --- | --- | --- | --- | --- | --- | --- | --- |
| **Parameter** | **Invasion**  **carcinoma** | **In situ carcinoma** | ***P*** | **Invasion carcinoma** | **In situ carcinoma** | ***P*** | **Invasion carcinoma** | **In situ carcinoma** | ***P*** |
| **Patients** | 733 (95) | 37 (5) |  | 142 (96) | 6 (4) |  | 93 (90) | 10 (10) |  |
| **Age, years (mean ± SD)** | 54.47±10.11 | 55.54±11.60 | 0.59 | 55.94±9.05 | 46.83±9.91 | 0.07 | 53.11±10.13 | 54.80±13.14 | 0.70 |
| **Lesion diameter, cm** | 2.44±1.10 | 2.70±2.20 | 0.48 | 2.45±1.08 | 3.88±1.93 | 0.13 | 2.94±1.60 | 4.04±3.14 | 0.30 |
| <=1 | 26 (3.55) | 9 (24.33) |  | 5 (3.52) | 0 |  | 1 (1.08) | 0 |  |
| 1-2 | 287 (39.15) | 8 (21.62) |  | 50 (35.21) | 0 |  | 26 (27.95) | 2 (20) |  |
| > 2 | 420 (57.30) | 20 (54.05) |  | 87 (61.27) | 6 (100) |  | 66 (70.97) | 8 (80) |  |

Note: SD, standard deviation. **P* < ·05.

**eTable 2** Performance of the proposed AI model in diagnosis of in situ and invasive carcinoma.

| **Performance Metric** | **Construction Set** | **Internal Test Set** | **Pooled External Test Set** |
| --- | --- | --- | --- |
| AUC (95% CI) | 0.964 (0.937-0.987) | 0.824 (0.682-0.938) | 0.788 (0.668-0.889) |
| ACC (95% CI) | 0.992 (0.983-0.997) | 0.784 (0.709-0.847) | 0.757 (0.663-0.836) |
| SENS (95% CI) | 0.838 (0.673-0.932) | 0.833 (0.365-0.991) | 0.700 (0.354-0.919) |
| SPEC(95%CI) | 1.000 (0.994 -1.000) | 0.782 (0.703-0.845) | 0.763 (0.662-0.843) |
| PPV(95%CI) | 1.000 (0.863-1.000) | 0.139 (0.052-0.303) | 0.241 (0.110-0.439) |
| NPV(95%CI) | 0.992 (0.982-0.997) | 0.991 (0.944-1.000) | 0.959 (0.878-0.989) |

Note: AI = Artificial Intelligence, 95% CI = 95% confidence intervals, ACC = accuracy, SENS = sensitivity, SPEC = specificity, PPV = positive predictive value, NPV = negative predictive value.

**eTable 3** Selected radiomics features and LASSO coefficients.

| **Category** | **Radiomics Features** | **Coefficients** | **Modality** |
| --- | --- | --- | --- |
| First-order statistics | lbp-2D_firstorder_10Percentile | 0.031243637 | Low-energy image |
| First-order statistics | lbp-2D_firstorder_Median | -0.016268991 | Low-energy image |
| Texture features | wavelet-LHH_glszm_ZoneEntropy | 0.032153298 | Low-energy image |
| Texture features | wavelet-HLL_glszm_ZoneEntropy | 0.023956414 | Low-energy image |
| Texture features | wavelet-HHH_glrlm_ShortRunLowGrayLevelEmphasis | -0.03875415 | Low-energy image |
| Texture features | wavelet-HLH_gldm_LargeDependenceHighGrayLevelEmphasis | 0.026825127 | Low-energy image |
| Texture features | wavelet-HLH_glszm_ZoneEntropy | 0.01572036 | Low-energy image |
| Texture features | wavelet-HLH_glrlm_ShortRunLowGrayLevelEmphasis | -0.017193908 | Low-energy image |
| Texture features | wavelet-LHH_glszm_SizeZoneNonUniformityNormalized | -0.02410295 | Low-energy image |
| Texture features | wavelet-HLL_glrlm_ShortRunLowGrayLevelEmphasis | -0.026875387 | Low-energy image |
| Texture features | wavelet-HHH_gldm_DependenceEntropy | 0.005000575 | Low-energy image |
| Texture features | wavelet-LHL_gldm_LargeDependenceLowGrayLevelEmphasis | 0.013248119 | Low-energy image |
| Texture features | wavelet-LHH_firstorder_Skewness | -0.009487831 | Low-energy image |
| Texture features | wavelet-LHL_firstorder_Skewness | -0.030213187 | Low-energy image |
| Texture features | wavelet-LLH_firstorder_Kurtosis | -0.037034843 | Low-energy image |
| Texture features | wavelet-LHH_glszm_SmallAreaLowGrayLevelEmphasis | 0.010015801 | Low-energy image |
| Texture features | wavelet-LLH_glszm_GrayLevelNonUniformity | 0.006573475 | Low-energy image |
| Texture features | wavelet-HLH_firstorder_Skewness | -0.012411982 | Low-energy image |
| Texture features | wavelet-LLH_glszm_SizeZoneNonUniformity | 0.002282752 | Low-energy image |
| Texture features | wavelet-HLH_glszm_SizeZoneNonUniformity | -0.027842206 | Low-energy image |
| Texture features | wavelet-HLL_gldm_DependenceVariance | -0.002979491 | Low-energy image |
| Texture features | wavelet-LHL_glszm_SizeZoneNonUniformity | -0.023038061 | Low-energy image |
| Texture features | wavelet-LHH_glszm_SizeZoneNonUniformity | -0.007559824 | Low-energy image |
| Texture features | wavelet-HLH_gldm_DependenceVariance | -0.002117247 | Low-energy image |
| Texture features | wavelet-HHH_glszm_SizeZoneNonUniformityNormalized | -0.018439061 | Low-energy image |
| Texture features | wavelet-HHL_gldm_DependenceEntropy | 0.007185972 | Low-energy image |
| Texture features | wavelet-HHL_firstorder_Skewness | 0.001662354 | Low-energy image |

**eTable 4** The performance of four radiomics models in construction set, internal and pooled external test sets.

| **Performance Metric** | **LR** | **SVM** | **KNN** | **DT** |
| --- | --- | --- | --- | --- |
| **Construction set** | | | | |
| AUC (95% CI) | 0.759 (0.734-0.789) | 0.758 (0.732-0.786) | 0.827 (0.806-0.848) | 0.678 (0.650-0.706) |
| ACC (95% CI) | 0.759 (0.733-0.783) | 0.756 (0.731-0.781) | 0.768 (0.743-0.792) | 0.630 (0.602-0.658) |
| SENS (95% CI) | 0.833 (0.806-0.857) | 0.815 (0.787-0.840) | 0.795 (0.766-0.821) | 0.612 (0.578-0.644) |
| SPEC (95% CI) | 0.554 (0.497-0.610) | 0.596 (0.539-0.650) | 0.694 (0.640-0.744) | 0.682 (0.626-0.732) |
| PPV (95% CI) | 0.837 (0.810-0.861) | 0.857 (0.820-0.870) | 0.877 (0.852-0.899) | 0.841 (0.809-0.868) |
| NPV (95% CI) | 0.547 (0.491-0.603) | 0.529 (0.485-0.592) | 0.552 (0.501-0.601) | 0.390 (0.349-0.432) |
| F1 score | 0.835 | 0.830 | 0.834 | 0.708 |
| **Internal test set** |  |  |  |  |
| AUC (95% CI) | 0.783 (0.703-0.853) | 0.758 (0.673-0.831) | 0.756 (0.662-0.836) | 0.671 (0.585-0.756) |
| ACC (95% CI) | 0.781 (0.716-0.836) | 0.718 (0.632-0.793) | 0.809 (0.731-0.873) | 0.634 (0.545-0.716) |
| SENS (95% CI) | 0.805 (0.732-0.863) | 0.729 (0.627-0.812) | 0.896 (0.813-0.946) | 0.635 (0.530-0.729) |
| SPEC (95% CI) | 0.690 (0.528-0.819) | 0.686 (0.506-0.826) | 0.571 (0.395-0.732) | 0.629 (0.449-0.780) |
| PPV (95% CI) | 0.905 (0.840-0.946) | 0.864 (0.766-0.927) | 0.851 (0.764-0.912) | 0.824 (0.715-0.900) |
| NPV (95% CI) | 0.492 (0.361-0.622) | 0.480 (0.339-0.624) | 0.667 (0.471-0.821) | 0.386 (0.263-0.524) |
| F1 score | 0.850 | 0.791 | 0.873 | 0.718 |
| **External test set** |  |  |  |  |
| AUC (95% CI) | 0.674 (0.593-0.759) | 0.658 (0.554-0.760) | 0.634 (0.550-0.727) | 0.669 (0.565-0.768) |
| ACC (95% CI) | 0.609 (0.521-0.692) | 0.737 (0.654-0.809) | 0.744 (0.662-0.816) | 0.677 (0.590-0.755) |
| SENS (95% CI) | 0.553 (0.452-0.650) | 0.874 (0.790-0.928) | 0.903 (0.825-0.950) | 0.680 (0.579-0.766) |
| SPEC (95% CI) | 0.800 (0.609-0.916) | 0.267 (0.130-0.462) | 0.200 (0.084-0.391) | 0.667 (0.471-0.821) |
| PPV (95% CI) | 0.905 (0.798-0.961) | 0.804 (0.716-0.870) | 0.795 (0.708-0.862) | 0.875 (0.778-0.935) |
| NPV (95% CI) | 0.343 (0.236-0.467) | 0.381 (0.190-0.613) | 0.375 (0.163-0.641) | 0.377 (0.251-0.521) |
| F1 score | 0.687 | 0.837 | 0.845 | 0.765 |

Note: LR = logistic regression, SVM = support vector machine, KNN = K-nearest neighbor, DT = decision tree, 95% CI = 95% confidence intervals, AUC = area under the receiver operating characteristic curve, ACC = accuracy, SENS = sensitivity, SPEC = specificity, PPV = positive predictive value, NPV= negative predictive value.

**eTable 5** The performance of four clinical models in construction set, internal and pooled external test sets.

| **Performance Metric** | **LR** | **SVM** | **KNN** | **DT** |
| --- | --- | --- | --- | --- |
| **Construction set** | | | | |
| AUC (95% CI) | 0.787 (0.760-0.813) | 0.770 (0.742-0.797) | 0.869 (0.851-0.887) | 0.858 (0.838-0.880) |
| ACC (95% CI) | 0.762 (0.738-0.787) | 0.754 (0.727-0.779) | 0.772 (0.746-0.797) | 0.821 (0.797-0.843) |
| SENS (95% CI) | 0.793 (0.763-0.820) | 0.783 (0.752-0.810) | 0.758 (0.726-0.787) | 0.861 (0.835-0.884) |
| SPEC (95% CI) | 0.679 (0.622-0.761) | 0.676 (0.619-0.728) | 0.811 (0.761-0.853) | 0.713 (0.657-0.763) |
| PPV (95% CI) | 0.870 (0.843-0.893) | 0.858 (0.840-0.891) | 0.916 (0.892-0.935) | 0.891 (0.866-0.911) |
| NPV (95% CI) | 0.546 (0.494-0.598) | 0.533 (0.481-0.585) | 0.552 (0.504-0.599) | 0.653 (0.598-0.705) |
| F1 score | 0.830 | 0.801 | 0.829 | 0.874 |
| **Internal test set** |  |  |  |  |
| AUC (95% CI) | 0.836 (0.770-0.895) | 0.817 (0.749-0.878) | 0.774 (0.691-0.852) | 0.776 (0.691-0.862) |
| ACC (95% CI) | 0.857 (0.800-0.903) | 0.842 (0.783-0.890) | 0.816 (0.755-0.868) | 0.842 (0.783-0.890) |
| SENS (95% CI) | 0.909 (0.849-0.948) | 0.890 (0.827-0.932) | 0.864 (0.797-0.912) | 0.890 (0.827-0.932) |
| SPEC (95% CI) | 0.667 (0.504-0.800) | 0.667 (0.504-0.800) | 0.643 (0.480-0.780) | 0.667 (0.504-0.800) |
| PPV (95% CI) | 0.909 (0.849-0.948) | 0.907 (0.845-0.946) | 0.899 (0.836-0.940) | 0.907 (0.846-0.946) |
| NPV (95% CI) | 0.667 (0.504-0.800) | 0.622 (0.465-0.758) | 0.563 (0.413-0.702) | 0.622 (0.465-0.758) |
| F1 score | 0.909 | 0.896 | 0.879 | 0.896 |
| **External test set** |  |  |  |  |
| AUC (95% CI) | 0.781 (0.709-0.841) | 0.790 (0.716-0.848) | 0.633 (0.547-0.722) | 0.699 (0.625-0.770) |
| ACC (95% CI) | 0.677 (0.590-0.755) | 0.744 (0.662-0.816) | 0.654 (0.567-0.734) | 0.677 (0.590-0.755) |
| SENS (95% CI) | 0.612 (0.510-0.705) | 0.903 (0.825-0.950) | 0.680 (0.579-0.766) | 0.738 (0.640-0.817) |
| SPEC (95% CI) | 0.900 (0.723-0.974) | 0.200 (0.084-0.391) | 0.567 (0.377-0.740) | 0.467 (0.288-0.654) |
| PPV (95% CI) | 0.955 (0.864-0.988) | 0.795 (0.708-0.862) | 0.843 (0.743-0.911) | 0.826 (0.730-0.894) |
| NPV (95% CI) | 0.403 (0.287-0.530) | 0.375 (0.163-0.641) | 0.340 (0.216-0.489) | 0.341 (0.206-0.507) |
| F1 score | 0.746 | 0.845 | 0.753 | 0.779 |

Note: LR = logistic regression, SVM = support vector machine, KNN = K-nearest neighbor, DT = decision tree, 95% CI = 95% confidence intervals, AUC = area under the receiver operating characteristic curve, ACC = accuracy, SENS = sensitivity, SPEC = specificity, PPV = positive predictive value, NPV = negative predictive value.

**eTable 6** The time for the AI model segment and analysis imaging compared to the time for radiologists.

|  | **Internal test set (n=196)** | **Pooled external test set (n=133)** |
| --- | --- | --- |
| **AI model** | 1.9h | 1.2h |
| **R4** | 5.1h | 3.9h |
| **R5** | 4.2h | 3.0h |

Note: AI = Artificial Intelligence, R = radiologist, h = hour.


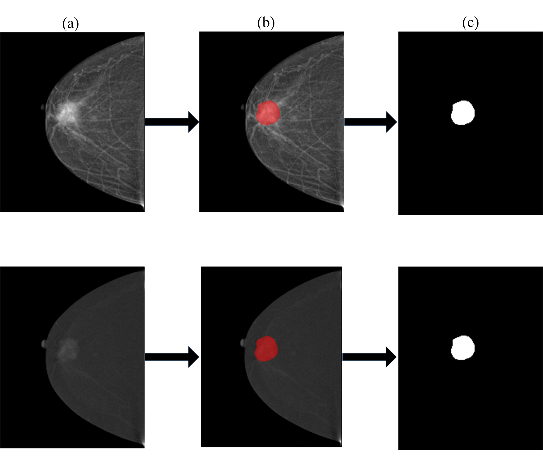


**eFigure 1** Segmentation process of the ROIs on the CEM images. (a) Low-energy (up) and recombined images (down) on the cranial caudal (CC) position. (b) ROIs delineated on the low-energy (up) and recombined (down) images. (c) Generated binary mask image.

**
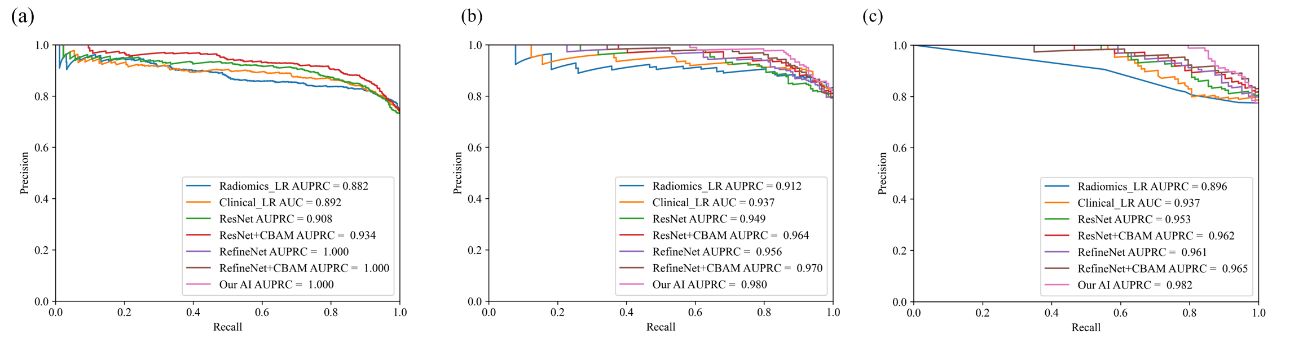
**

**eFigure 2** PRCs of the different models in the construction set (a), internal test set (b), and pooled external test set (c).


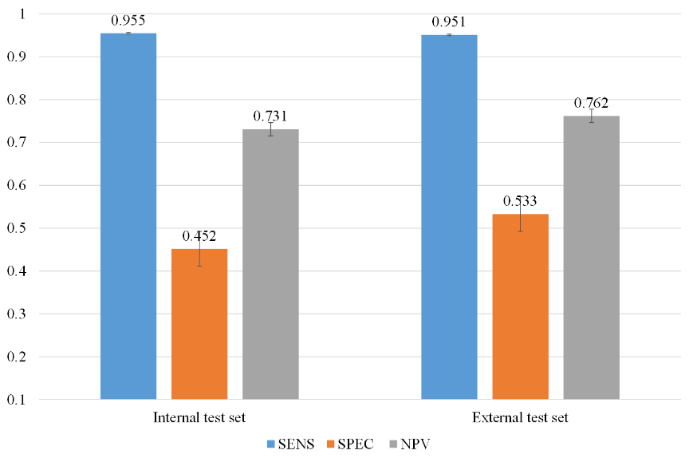


**eFigure 3** The performance of the AI model for 95% sensitivity threshold on internal and pooled external test sets.


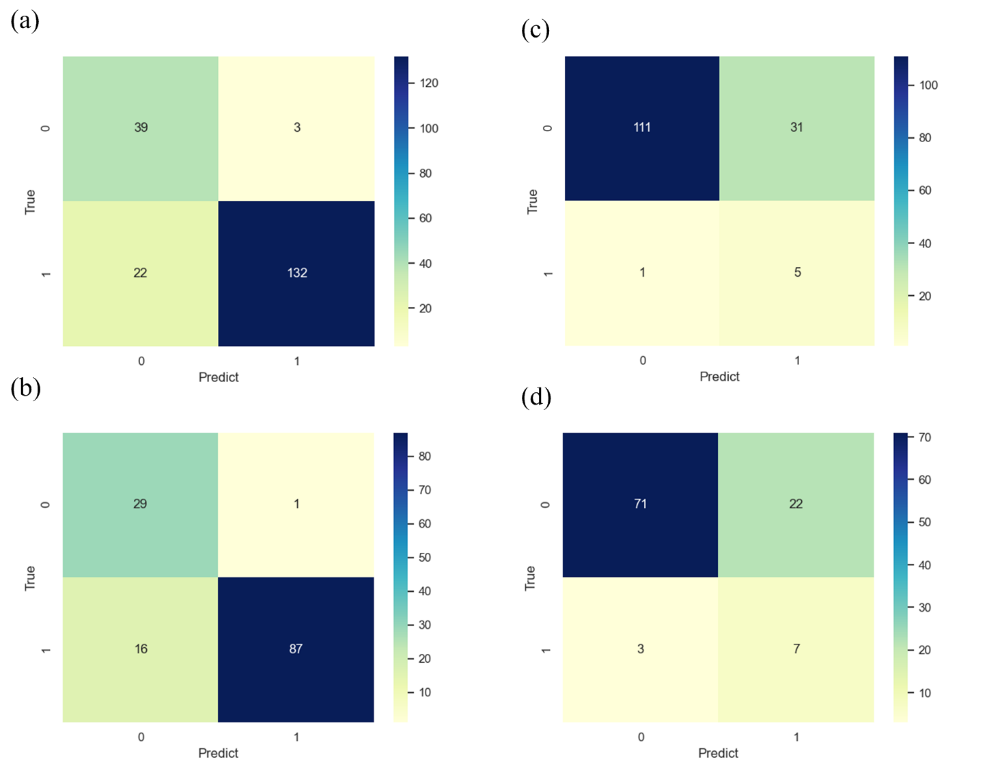


**Figure 4** Confusion matrix of our AI model for diagnosis of benign and malignant of breast lesions in the internal test set (a) and pooled external test set (b) and for diagnosis of in situ and invasive carcinoma in the internal test set (c) and pooled external test set (d).

**eFigure 5** Loss (a) and accuracy (b) curve of our AI model in the training set (red) and validation set (blue). The accuracy and loss are plotted for each epoch. The accuracy curves of the construction set are represented by the rising red and blue curves, respectively. The loss curves on the construction set are represented by the descending curves.

**
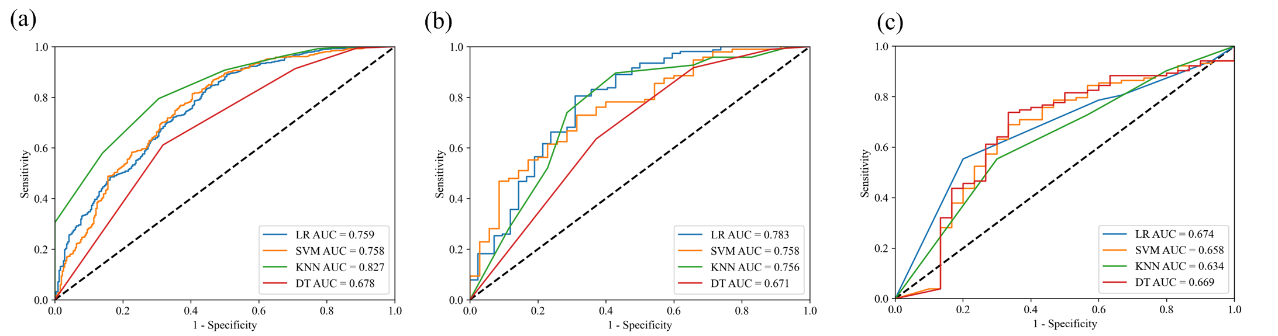
**

**eFigure 6** ROC curves of the radiomics models in the construction set (a), internal test set (b) and pooled external test set (c). AUC, area under the ROC curve

**
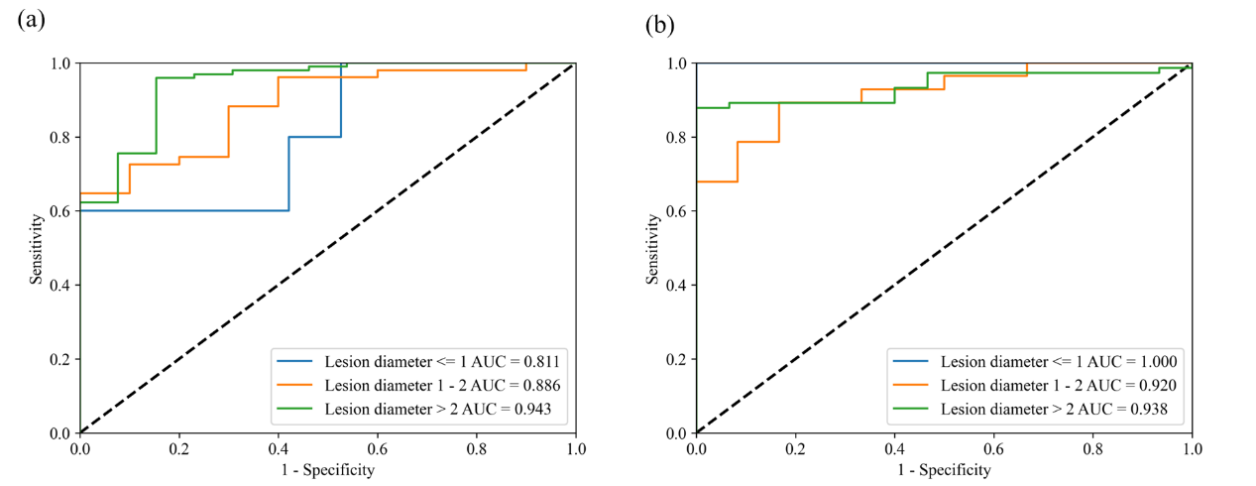
**

**eFigure 7** ROC curves of the our AI model in different lesion diameter subgroups on the internal test set (a) and pooled external test set (b).
